# A Simple Method for Sample Preparation to Facilitate Efficient Whole-Genome Sequencing of African Swine Fever Virus

**DOI:** 10.3390/v11121129

**Published:** 2019-12-06

**Authors:** Ferenc Olasz, István Mészáros, Szilvia Marton, Győző L. Kaján, Vivien Tamás, Gabriella Locsmándi, Tibor Magyar, Ádám Bálint, Krisztián Bányai, Zoltán Zádori

**Affiliations:** 1 Institute for Veterinary Medical Research, Centre for Agricultural Research, Hungária krt. 21, 1143 Budapest, Hungary; meszaros.istvan@agrar.mta.hu (I.M.); marton.szilvia@agrar.mta.hu (S.M.); kajan.gyozo@agrar.mta.hu (G.L.K.); tamas.vivien@agrar.mta.hu (V.T.); magyar.tibor@agrar.mta.hu (T.M.); banyai.krisztian@agrar.mta.hu (K.B.); zadori.zoltan@agrar.mta.hu (Z.Z.); 2 Veterinary Diagnostic Directorate, National Food Chain Safety Office, Tábornok u. 2, 1149 Budapest, Hungary; LocsmandiG@nebih.gov.hu (G.L.); balintad@nebih.gov.hu (Á.B.)

**Keywords:** African swine fever virus, ASFV, whole genome sequencing, whole genome amplification, NGS, Illumina, Hungarian ASFV strain, DNAse treatment, whole genome amplification

## Abstract

In the recent years, African swine fever has become the biggest animal health threat to the swine industry. To facilitate quick genetic analysis of its causative agent, the African swine fever virus (ASFV), we developed a simple and efficient method for next generation sequencing of the viral DNA. Execution of the protocol does not demand complicated virus purification steps, enrichment of the virus by ultracentrifugation or of the viral DNA by ASFV-specific PCRs, and minimizes the use of Sanger sequencing. Efficient DNA-se treatment, monitoring of sample preparation by qPCR, and whole genome amplification are the key elements of the method. Through detailed description of sequencing of the first Hungarian ASFV isolate (ASFV_HU_2018), we specify the sensitive steps and supply key reference numbers to assist reproducibility and to facilitate the successful use of the method for other ASFV researchers.

## 1. Introduction

African swine fever virus (ASF) is a devastating disease affecting Sus scrofa; it infects both domesticated pigs and wild boars. The ASF virus (ASFV) was most probably transmitted from its natural hosts, warthogs (*Phacochoerus* spp.), bushpigs (*Potamochoerus* spp.) and soft ticks of the genus Ornithodoros to domestic pigs (*Sus scrofa domesticus*) in southeast Africa [1,2]. The virus is endemic in the Sub-Saharan region, where viral reservoir is maintained by a sylvatic cycle between the soft ticks and its natural hosts [3,4]. In countries with temperate climate, mainly direct contact between domestic pigs and wild boars, and indirect contact facilitated by human activity sustain the infectious cycle [4,5]. First transmission of ASFV to domestic pigs was reported in East Africa in 1921 [3]. The virus was introduced into Europe in several successive waves; genotype I virus spread to the Southern Europe in the 1950’s and 1960’s, while the well documented emergence of genotype II ASFV occurred in Georgia in 2007 [6]. Since 2017 the virus has continuously spread westward in Eastern Europe, reaching Hungary and Belgium, then in 2018 it arrived to China, and by now it became arguably the biggest economic and animal health threat to the swine industry of the world [7,8].

ASFV is an enveloped virus with a large (170−190 kilo base pair) double stranded, covalently closed, linear DNA genome, which contains around 200 open reading frames. The genome organization of the virus is reminiscent that of the poxviruses: it consists of a more conserved central area (approx. 125 kb) flanked by two variable regions (38-47 kb and 13–16 kb, respectively) at the ends of the genome [6,9,10].

The ASFV has a high genetic and antigenic diversity. Determined by the p72 protein (B646L), so far 24 genotypes have been identified, while based on hemadsorption inhibition at least 8 serotypes are recognized [11,12]. All genotypes occur in Africa, from which genotype I of West African origin caused outbreaks in the European, Caribbean, and South and Central American regions before 2007. However, the pandemic that originated from Georgia was caused by the emergence of a genotype II virus of East African origin [13]. Comparison of the complete sequences of the original Georgia 2007/1 genome and the Polish ASFV/Pol/2015/Podlaskie genome isolated eight years later revealed only 95 nucleotide differences scattered in the 190 000 bp genome, suggesting a relatively slow in vivo evolution of this genotype II ASFV in pigs [14].

Vaccine development is hampered by the lack of detailed knowledge about viral virulence and immunological factors influencing the outcome of infection of and immune response to ASFV. Currently there are no available continuous cell lines supporting the replication of ASFV field isolates without major genetic makeovers. Adaptation of ASFV to established cell lines usually leads to genome destabilization and loss of ability to replicate in macrophages in vitro and in vivo. However, ASFV can be isolated and replicated in primary macrophages without obvious genetic alterations [15,16].

So far, only 69 near complete genome sequences (including unverified ones and sequences from patents) have been deposited into the GenBank (status 31.10.2019) [17] despite the obvious animal health significance of the virus. Attempts to produce additional complete sequences of biologically relevant ASFV strains are frequently thwarted by technical problems, such as difficulty in achieving sequencing grade ASFV DNA and applying the most suitable next generation sequencing (NGS) method for their production. To facilitate much needed epidemiological investigations, advance research and vaccine development, it would be expedient to have a simple and reproducible method for full genome sequencing of the ASFV.

In this paper we present a protocol that was successfully used to sequence the first Hungarian isolate of ASFV and in our opinion fulfills the aforementioned criteria.

## 2. Materials and Methods 

### 2.1. PAM Preparation and Culture

Porcine alveolar macrophages (PAMs) were prepared according to the OIE Manuals [18] and were stored in RPMI-1640 medium containing 30% bovine serum and 10% DMSO at −72 °C. PAMs were cultured in PAM culturing media (RPMI-1640 medium supplemented with 10% (*v*/*v*) fetal bovine serum, 100 U/mL penicillin, 100 μg/mL streptomycin, and 2 mM L-glutamine (Sigma-Aldrich, Saint Louis, MO, USA) at 37 °C and in 5% CO_2_.

### 2.2. Virus Isolation and Immunofluorescence Detection

Tissue homogenates of an ASFV-infected carcass were prepared with Tissue Lyser (QIAGEN, Hilden, Germany) in PAM culturing medium sterile-filtered with 0.2 μm Acrodisc syringe filter (Pall Corporation, NY, USA) and serially diluted in half-log steps. 2 × 10^4^ PAM cells (100 μL) were plated in 96-well plates, incubated for 16 h at 37 °C, and infected with 10 μL of the diluted tissue homogenates. After three days of incubation, the supernatant was removed and stored at −72 °C, while cells were permeabilized by 1% Triton-X and fixed in 3% formaldehyde solution. Infected cells were visualized by anti-ASFV polyclonal sera and Goat Anti-Swine IgG (H+L) CF488A (Biotium, Fremont, CA, USA) secondary antibody. Positive cells were detected under an Axio Observer D1 inverted fluorescence microscope (Carl Zeiss Ag. Oberkochen, Germany). 

### 2.3. Viral Stock Preparation

PAM cells (10^5^ cells in 1ml PAM culturing media) were infected with 30 μL high-titre ASFV-infected PAM cell supernatant in several wells of a 24-well plate and incubated at 37 °C. At 72 h post infection (hpi), the supernatant was removed, aliquoted, and stored at −72 °C.

### 2.4. DNAse Treatment 

The media of ASFV-infected cells were collected at 72 hpi and centrifuged at 13 000 g for 3 min to get rid of cellular debris. Subsequently, to 100 µL of the supernatant 100 µL DNase I solution containing 20 µL 10× FastDigest buffer (Thermo Fischer, Waltham, MA, USA), 40 mM MgCl_2_ and 1.5 µL of the DNase I (50 U/µL) (Thermo Fischer Scientific, Waltham, MA, USA) was added. The samples were incubated at 37 °C for 1 h, after which 10 µL 0.5 M EDTA was added to stop the reaction.

### 2.5. DNA Purification

Viral DNA was purified with the High Pure Viral Nucleic Acid Kit (Roche, Basel Switzerland) following the manufacturer’s recommendations. In brief, 200 µL binding buffer and 50 µL proteinase K were added to 200 µL sample, mixed, and incubated at 72 °C for 10 min. An additional 100 µL binding buffer was added to the samples, mixed and transferred to the nucleic acid-binding membranes. After centrifugation at 8000× *g* for 1 min, the membranes were washed first with 500 µL inhibitor removal buffer and twice with 450 µL wash buffer. The DNA was eluted by 50 µL elution buffer.

### 2.6. Quantitative PCR

ASFV specific dual quantitative PCR (qPCR) was executed by Virotype ASFV PCR Kit (Qiagen, Hilden, Germany) according to the manufacturer’s recommendation.

### 2.7. Aspecific DNA Amplification

The viral DNA was amplified using the REPLI-g Mini Kit (Qiagen, Hilden, Germany), following the manufacturer’s protocol. First, 5 µL denaturing buffer was added to 5 µL viral DNA sample and incubated at room temperature for 3 min. After that 10 µL neutralizing buffer and 30 µL master mix (containing 29 µL REPLI-g Reaction Buffer and 1 µL REPLI-g Mini DNA polymerase) were mixed with the denatured sample. The tubes were incubated at 30 °C for 16 h, then the polymerase was inactivated by heating up to 65 °C for 3 min.

### 2.8. Amplified DNA Clean Up

REPLI-g samples were purified using the NucleoSpin Gel and PCR clean-up Kit (Macherey-Nagel Düren, Germany). Briefly, 200 µL NTI buffer was added to 50 µL of the sample. After mixing, the solution was loaded to the spin column and centrifuged at 11,000× *g* for 1 min. The column was washed first with 500, then with 200 µL NT3 buffer. The remnant of the wash buffer was removed by centrifugation at 11,000× *g* for 1 min. The DNA was then eluted in 20 µL elution buffer, and its concentration was measured with NanoDrop 2000 (Thermo Fischer Scientific, Waltham, MA, USA).

### 2.9. IonTorrent Sequencing

A total of 100 ng of DNA was subjected to enzymatic fragmentation using the reagents supplied in the NEBNext Fast DNA Fragmentation & Library Prep Set for Ion Torrent kit (New England BioLabs, Hitchin, United Kingdom) according to the manufacturer’s instructions with slight modifications. In brief, 8 µL of DNA was mixed with 1 µL of NEBNext DNA Fragmentation Reaction buffer, 0.5 µL MgCl_2_ (using a 10 mM stock), and 0.75 µL NEBNext DNA Fragmentation Master Mix. The mixture was incubated at 25 °C for 20 min, then at 70 °C for 10 min. The adaptor ligation was performed using reagents from the same kit, whereas barcoded adaptors were retrieved from the Ion Xpress Barcode Adapters (Thermo Fischer Scientific, Waltham, MA, USA). Reaction components were used at a reduced volume: 2 µL T4 DNA Ligase Buffer for Ion Torrent, 2 µL barcode adapter mixture, 0.5 µL *Bst* DNA Polymerase and 2 µL T4 DNA Ligase were combined with the fragmentation reaction mixture and nuclease-free water to obtain a final volume of 20 µL. Adapter ligation was performed at 25 °C for 15 min, terminated at 65 °C for 5 min. After cooling on ice slurry, 2.5 µL of Stop Buffer was added to the mixture. The barcoded library DNA samples were purified using the Gel/PCR DNA fragments extraction kit (Geneaid Biotech, Ltd., Taipei, Taiwan) according to the manufacturer’s instructions. The eluted DNA libraries were then run on 2% E-Gel SizeSelect II Agarose (Invitrogen, Carlsbad, CA, USA). Products between 300 and 350 bp were directly used in the PCR mixture of the NEBNext Fast DNA Fragmentation & Library Prep Set for Ion Torrent kit (New England BioLabs, Hitchin, United Kingdom) without further purification. 

Library amplification was made in a total volume of 50 µL (the reaction mixture consisted of 15 µL sample, 7.5 µL H_2_O, 25 µL enzyme mix, and 2.5 µL primer), the heat profile included an initial denaturation at 98 °C for 30 s, followed by 12 amplification cycles (98 °C for 10 s, 58 °C for 30 s, 72 °C for 30 s) and terminated at 72 °C for 5 min. The products were purified using the Gel/PCR DNA fragments extraction kit (Geneaid). The library DNA was eluted in nuclease-free water and quantified fluorometrically on Qubit 2.0 equipment using the Qubit dsDNA BR assay kit (Invitrogen, Carlsbad, CA, USA). Subsequently, the library DNA was diluted to 10 to 14 pM, then clonally amplified by emulsion PCR. This step was carried out according to the manufacturer’s instructions using the Ion PGM Hi-Q View OT2 Kit on an Ion OneTouch 2 instrument. Enrichment of the templated beads (on an Ion OneTouch ES machine) and further steps for pre-sequencing setup were performed according to the 200-bp protocol of the manufacturer. The sequencing protocol recommended for the Ion PGM sequencing kit on a 316 chip was strictly followed.

### 2.10. Illumina Sequencing

Illumina^®^ Nextera XT DNA Library Preparation Kit (Illumina, San Diego, CA, USA) and Nextera XT Index Kit v2 Set A (Illumina, San Diego, CA, USA) were used to prepare Illumina specific libraries. DNA samples were diluted to 0.2 ng/μL in nuclease-free water (Promega, Madison, WI, USA) in a final volume of 2.5 μL. For the tagmentation reaction, 5 μL Tagment DNA (TD) buffer with 2.5 μL AmpliconTagment Mix (ATM) were used. Next, the samples were incubated at 55 °C for 6 min, using the GeneAmp PCR System 9700 (Applied Biosystems, Foster City, CA, USA). The samples were then allowed to cool to 10 °C before the immediate addition of 2.5 μL of the Neutralize Tagment (NT) buffer. Neutralization was performed for 5 min at room temperature. A total of 7.5 μL of the Nextera PCR Master Mix (NPM) was combined with i5 and i7 index primers (2.5 μL of each primer per well) and added to the tagmented DNA sample. The index primers were incorporated into library DNA via 12 PCR cycles (each cycle consisted of the following steps: 95 °C for 10 s, 55 °C for 30 s, followed by 72 °C for 30 s). Following the PCR cycles, the samples were held at 72 °C for 5 min and then at 10 °C. The PCR products were purified using Gel/PCR DNA Fragments Extraction Kit (Geneaid Biotech Ltd., Taipei, Taiwan). The concentration of the purified DNA samples was quantified with Qubit 2.0 equipment using Qubit dsDNA HS Assay Kit (Thermo Fischer Scientific, Waltham, MA, USA). Library DNAs were pooled and denatured. Denatured library pool at a final concentration of 1.5 pM was loaded onto a NextSeq 500/550 Mid Output flowcell and sequenced using an Illumina^®^ NextSeq 500 sequencer (Illumina, San Diego, CA, USA).

### 2.11. Mapping and Assembly

Sequence reads obtained by NGS were trimmed by using Geneious Prime 2019.0.3 (Biomatters Ltd., Auckland New Zealand). The reads were mapped against the pig genome (Sscrofa11.1, GenBank assembly accession: GCF_000003025.6) to determine and eliminate host DNA contaminations. The purified reads were assembled to the viral genome by mapping these to the sequence ASFV Belgium 2018/1 [19] strains as reference. The sensitivity of mapping was set to medium with three iteration. The graphic representation of coverage and gene predictions were made using Geneious Prime 2019.0.3.

### 2.12. Sanger Sequencing

Three regions were amplified using Primestar GXL kit with GC buffer (Takara Bio Inc., Japan), in 25 μL final volume with 1 μL of purified ASFV DNA according to the manufacturer’s instructions. The amplification was performed using the following PCR program: 98 °C 30 s, 30 × (98 °C 10 s, 60 °C 15 s, 68 °C 1 min), 68 °C 2 min. The primer pairs used for the amplification of the different fragments are listed in Table 1. The same primers were used for Sanger sequencing by BaseClear B.V. (Leiden, Netherlands). Sequences of the amplicons were well defined from both directions upstream of the poly C/G on each fragment, while downstream of the C/G tracts sequence slippage was detected in each case. The size of the poly C/G tracts and the actual sequence around them were determined by comparing and analyzing the reads of the opposing strands.

### 2.13. Phylogenetic Analyses

Two phylogenetic analyses were conducted: first, ASFV complete genomes were compared, then genotype I and II strains were picked from this analysis together with an outgroup strain for new tree inference. The transposed 5′ genomic end of strain Estonia 2014 (LS478113) [20] was trimmed from the genome to ease aligning. For phylogenetic tree inference, multiple alignments were conducted using MAFFT [21] with the legacy gap penalty, and phylogenetic calculations were performed using RAxML-NG v0.9.0 [22] based on alignments edited in trimAl v1.3 [23]. Evolutionary model selection was performed using ModelTest-NG v0.1.5 [24] and the generalized time reversible (GTR) model had the highest probability combined with discrete Gamma rate categories (+*G*) and the proportion of invariant sites (+I) [25]. The robustness of the trees was determined with a non-parametric bootstrap calculation using 1000 repeats. Phylogenetic trees were visualized using MEGA 7 [26], bootstrap values are given as percentages if they reached 75%.

## 3. Results and Discussion

ASFV_HU_2018 was isolated from homogenized organ samples of a wild boar carcass on PAM cells. The isolated virus was assigned to be sequenced to determine its genetic makeup and to gain molecular epidemiological data that might help identify its origin. However, following published NGS-based general viral metagenome sequencing protocols we were unable to assemble the complete ASFV genome sequence. The main reason of the failure was that viral sequences represented only a minor fraction (<0.5%) of the total NGS reads [27] 

Similar problems were reported at the sequencing of other ASFV isolates and different methodical approaches were pursued to overcome the difficulties [28,29,30,31,32]. These include animal infections [33], the complicated purification of the virus from animal blood [14,33], the enrichment of the virus by ultracentrifugation or that of the viral DNA by ASFV-specific PCR in the samples, and the excessive use of Sanger sequencing [14,29,30].

The majority of the “ASFV sequencing papers” that made their virus/host genome read ratio available reported very low percentage (0.5–1.0%) [27,34] of viral reads that resulted in low coverage and consequently, the frequent use of specific PCR-Sanger sequencing (SPSS) to patch up ambiguous, uncovered or poorly covered genome stretches. Regrettably, large-scale use of SPSS can substantially increase the time and money spent to achieve the complete sequence of a near 200 Kbp virus.

Wen and coworkers [32], gave up altogether the random “shotgun” approach of NGS and applied a whopping 86 overlapping specific PCRs (and sequenced the amplicons by NGS) to complete an ASFV genome after having very low percentage viral reads in their samples. This extreme example makes obvious that the full advantage of NGS for ASFV sequencing can only be exploited if a high percentage of the sample DNA derives from the viral sequence.

Having considered the aforementioned facts, our goal was to develop a simple and reliable NGS-based ASFV sequencing protocol, in which animal housing or ultracentrifugation are not involved, ASFV-specific PCRs can be minimized and can be executed in most of veterinary BSL3 laboratories using standard equipment. To achieve this, we concentrated on enriching the ASFV genome and decreasing the contaminating host genome in the input DNA samples using the simplest tools available to us.

### 3.1. Maximizing Viral DNA Content

**Figure 1:**
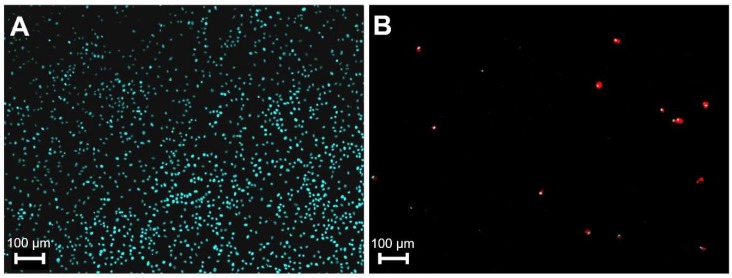
The effect of African swine fever virus (ASFV) infection on primary macrophages at 72 h p.i. Cells were mock infected (**A**) or infected (**B**) with MOI of 3 of ASFV. The nuclei of the cells were visualized by Hoechst 33342 reagent (blue), infected cells (red) were detected by ASFV positive sera and CF488 labelled anti-pig secondary antibodies. Pictures were colored by computer.

To maximize viral titer, ASFV was propagated in PAM cells, and the dynamics of infected cells was monitored by immunofluorescence at 24, 48, and 72 hpi. The infection rate and attached cell number at 24 and 48 hpi varied greatly (0.5–20% and 15–60%, respectively), even when the same cell and virus lots were used in parallel infections. However, quasi independently of the 24 hpi infection rate usually 90% of the cells lysed at 72 hpi and ~90% of the remaining attached cells proved to be infected (Figure 1). These observations indicated that the supernatants of the infected cells had to be collected at 72 hpi. They were centrifuged to get rid of contaminating cells and cellular debris and used for total DNA extraction. For DNA quantification, the Virotype ASFV PCR Kit (Qiagen, Hilden, Germany) was chosen as it allowed the simultaneous detection of the ASFV DNA and the contaminating swine genome by dual PCR. C_t_ values (~22 and ~29 for ASFV and pig DNA, respectively) indicated high molar ratio of the ASFV genome in the average samples. However, our ultimate goal was to get a high sequence read ratio between the virus and the host and this can be only achieved by a high mass ratio between the two genomes in the NGS input sample. Knowing the C_t_ values and size of the host and the ASFV genomes allowed us to roughly calculate the mass ratio of the two genomes. For example, assuming equally efficient amplifications and taking into consideration the size difference of the host (2.5 × 10^9^ bp of the haploid genome) and the ASFV (2 × 10^5^ bp) genomes, at least ~13.6 cycle difference (2.5 × 10^9^/2 × 10^5^ = 1.25 × 10^4^ = 2^13.6^) would be expected in a sample which contains equal mass of viral and host DNA. However, this assessment certainly underestimates the C_t_ difference measured in such a sample, as the kit is optimized to reduce the effectiveness of the host-specific PCR, to increase the sensitivity of the viral PCR [35]. Nonetheless, following the rationale presented above, the mass ratio of the viral and the contaminating host DNA can be calculated with the formula (2 × 10^5^/2^y^)/(2.5 × 10^9^/2^z^) × 100 where y and z are C_t_ values measured in the two channels of the dual PCR, respectively. In our specific case (2 × 10^5^/2^21.6^)/(2.5 × 10^9^/2^29.1^) × 100 = ~1.44%. Thus, relatively high C_t_ numbers (~29) measured in the second channel lagging behind with only 7–8 C_t_ values of the virus-specific channel in the reality indicated very high swine genome contents (>98.56%).

### 3.2. Minimizing Contaminating Host Genome DNA

DNAse treatment and ultracentrifugation are widely used to get rid of contaminating host DNA from ASFV samples [14,29,30,34]. Since ultracentrifuges are expensive and far from being standard appliances in veterinary BSL3 laboratories, we concentrated on increasing the effectiveness of the DNAse treatment and monitoring its result.

**Figure 2:**
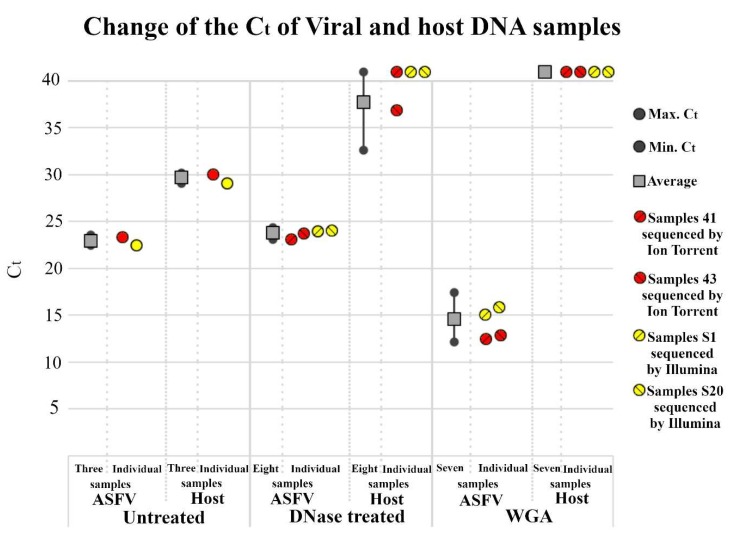
Host and viral DNA content of differently treated ASFV samples. Quantitative dual PCR was executed by Virotype ASFV PCR Kit. C_t_ values of individual and averaged samples represented by colored circles and grey box respectively. Averages were calculated from numbers of samples indicated on X axis. Gray circles indicate minimum and maximum values. C_t_ values higher than 40 (undetectable host DNA) are represented by 41. Whole genome amplification (WGA) was executed by REPLI-g Mini Kit.

Bovine serum-supplemented culture medium with cell lysate content is a complex protein solution with a potential to inhibit DNAse I activity through bivalent cation and DNA binding [36,37]. To ensure effective DNAse I treatment, the culture medium was diluted two-fold in a nuclease buffer containing added MgCl_2_. After 1-h incubation with 75U DNAse-I, the reaction was stopped with EDTA and viral nucleic acid was purified. In most cases, minimal increase (C_t_ +1-2) was detected between the C_t_ values of untreated and DNAse-treated samples indicating that a substantial part (25–50%) of viral DNA in the supernatant packed into DNAse-I resistant virions. The amount of host DNA on the other hand decreased markedly, however, the extent of reduction varied from sample to sample as indicated by the broad range of increases in the C_t_ value (4 to >13) in the second channel (Figure 2). These observations indicate the complexity and unpredictable nature of the virus-containing cell lysate and highlight the importance of the qPCR monitoring for the selection of the appropriate samples.

The addition of EDTA to inactivate the DNAse before DNA purification also seemed to be crucial. Omission of this step resulted in complete loss of the viral DNA in the following DNA purification step (data not shown), which suggests that inactivation of high concentrations of DNAse I is not a rapid process in the binding buffer of the applied purification kit (surprisingly, supplier does not indicate EDTA content) [38].

### 3.3. Nonspecific Amplification of the Viral DNA

To increase the absolute amount of DNA for the following NGS sample preparation protocols, whole genome amplification (WGA) was performed by using the REPLI-g Mini Kit. WGA was expected to sustain the original high viral/host DNA ratio by randomly and evenly multiplying host and viral DNA. Purified DNA samples with the highest viral content (viral C_t_ ~23, host C_t_ >40) were chosen for the reaction. After the WGA reaction qPCR revealed a ~1000-fold increase in the viral DNA content in the reaction tube (C_t_ ~23 vs. C_t_ ~14), while the host DNA remained undetectable (Figure 2). DNA from the WGA reaction was purified with the NucleoSpin Gel and PCR clean-up kit and the purification resulted in 0.3–0.8 µg DNA/reaction that were used for Illumina and Ion Torrent NGS.

### 3.4. NGS Sequencing of the ASFV Genome

In the last few years, Illumina and Ion Torrent systems became the most frequently used NGS platforms for whole genome sequencing of microorganisms. To find the most effective solution for ASFV sequencing [39], we compared the two methods by running two ASFV samples on each platform.

Analysis of the sequence data revealed that the number of viral reads exceeded the reads of the contaminating nucleic acids in all four samples (Table 2). This finding verified that the DNAse I treatment together with the applied monitoring procedure is in fact able to warrant that the majority of the DNA in the ASFV samples originate from the viral genome.

Genomes were assembled by mapping the viral reads to the sequence of ASFV Belgium 2018/1 [19] strains as reference by using the Geneious Prime 2019.0.3 program. Numerous (~131) undefined nucleotides, mainly in the form of single and double nucleotide insertions/deletions (indels) were found in homopolymer tracts of the assembled genome of the Ion Torrent platform-sequenced samples (minimum coverage 35) compared to the reference sequence. The high number of ambiguous reads made the usefulness of the Ion Torrent platform questionable for ASFV genome sequencing and it was omitted from further investigations.

The number of undefined nucleotides were much less frequent in the Illumina platform output. After processing the data of one sample (~7 million viral reads), they were restricted to the three “longest” homopolymer regions (14224–14236 (13C), 15665–15680 (16C), 19991–20001 (11G)) containing more than 10 C/G nucleotides. The sequence ambiguities of these regions could not even be resolved by processing all viral reads of the two Illumina samples (around 14 million viral reads). Thus, to determine the accurate sequence of the ASFV_HU_2018 (Accession number: MN715134), these three regions had to be sequenced by the Sanger method. The use of these SPSS indeed allowed completing the full sequence of the isolate.

Short of these three poly C/G tracts, the Illumina reads gave an even coverage for most of the genome in both samples (Mean coverage: 2692.4 with Std Dev: 1897.6 and 2557 with Std Dev: 1624.9) except for the terminal regions where viral reads were underrepresented.

**Figure 3:**
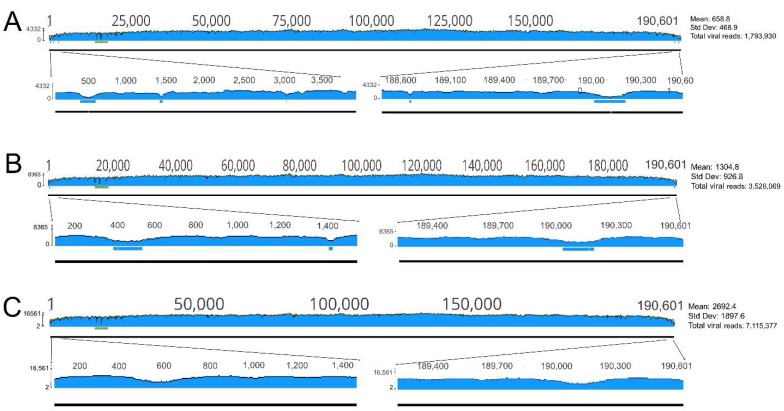
Graphical visualization of the nucleotide coverage values of the ASFV genome in sample S20. Horizontal blue bars represent regions with unsatisfying (<10) coverage in the terminal regions. Horizontal green bar labels the region with the three unresolvable C/G tracts. Numbers on the vertical scale indicate minimum and maximum coverage values. (**A**), data from a single channel; (**B**), data from two channels; (**C**) data from four channels.

Handling the data of only one channel (~1.8 million reads) still supplied very good coverage on most of the genome and resulted in five short nucleotide stretches (spanning altogether ~440 nucleotides) with unsatisfying coverage (<10) within the two terminal regions (1–3200 and 188,800–190601) that impeded the determination of the exact sequence (Figure 3). Although processing the data of two (3.6 million reads) or three (5.4 million reads) channels decreased the extension of the shortage of reads, it still left nucleotides unsatisfyingly (<10) covered in these 5 regions (Figure 3). The lower coverage of the termini most probably comes from the reduced amplification of these regions by the REPLI-g Mini Kit. The termini of the virus are covalently closed and contain inverted repeats (ITR) that facilitate the quick rehybridization of the template strands. Thus, the ITRs can impede the annealing of the random primers to these regions that leads to below average amplification and the underrepresentation of these regions in the sequence reads.

In any case, it seems that the generation of around 7 million reads and 3 SPSS are needed to assemble the complete sequence of an ASFV isolate with our protocol if we want to minimize the number of SPSS. The significant decrease of the NGS reads necessitate the increase of SPSS in the terminal regions.

### 3.5. Analysis of the Sequence

Comparison of the ASFV_HU_2018 sequence to the ASFV Belgium 2018/1 sequence resulted in only 22 nucleotide mismatches at 15 sites; the majority of these (9 sites) being mutations in non-coding regions. Approximately the same number of differences (19 mismatches at 14 sites) was found between the ASFV_HU_2018 and the China/2018/AnhuiXCGQ isolate [32] (Table 3). This relatively low number of differences provide indirect evidence for the good quality of sequencing in the different labs.

**Figure 4:**
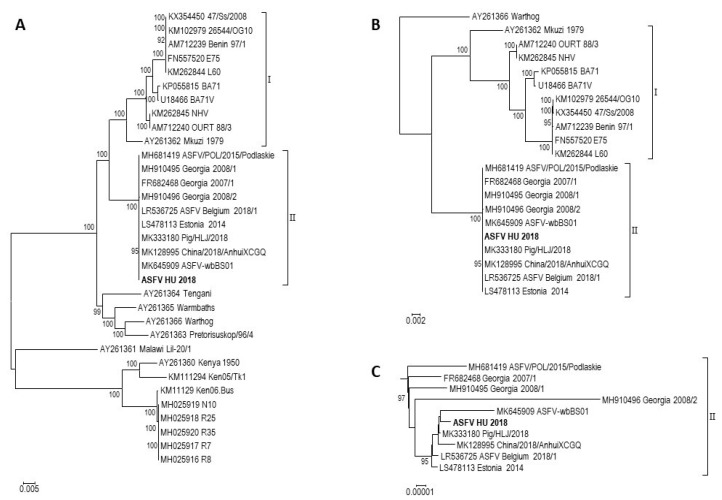
Phylogenetic analyses of complete ASFV genome sequences. The sequenced ASFV HU 2018 strain is highlighted using bold letters. Strains are represented using their NCBI Nucleotide accession numbers and their strain names. Genotypes I and II are marked. (**A**). Phylogenetic analysis of different ASFV genotypes. The tree was rooted on the midpoint. (**B**). Phylogenetic analysis of genotype I and II ASFV strains. The tree was rooted using strain Warthog as outgroup. (**C**). The subtree of genotype II strains shown separately from the phylogenetic analysis of genotype I and II ASFV strains.

It is appealing that the majority of the otherwise small differences are located in non-coding regions (9 and 7 sites, respectively). Considering that around 85% of the ASFV genome codes proteins and assuming random mutation distribution, the majority of the mutations should be localized in the coding regions (unless there is no purifying selection). This may suggest such a selection pressure on the viral proteins that sustains not only their protein but also their nucleotide sequences. Phylogenetic analysis of the ASFV complete genomes also support high genetic stability of the genotype II viruses. Interestingly, it reveals considerably less evolutionary distance among genotype II viruses than genotype I viruses (Figure 4). For example, the two most divergent genotype I viruses (Mkuzi 1979 [AY261362] and BA71V [U18466]) show ~90% nucleic acid identity (without the large deletions in BA71V) while the two most different genotype II ASFVs (2008/1 [MH910495] and Estonia 2014 [LS478113]) are almost 99.9% identical. This raises the question of whether genotype II viruses evolve at a lower speed in pigs than type I viruses do. However, the different evolutionary time of the two genotypes in pigs and the improving reliability of the sequencing technology since the emergence of ASFV makes the question difficult to answer and it requires further research.

## 4. Conclusions

**Figure 5:**
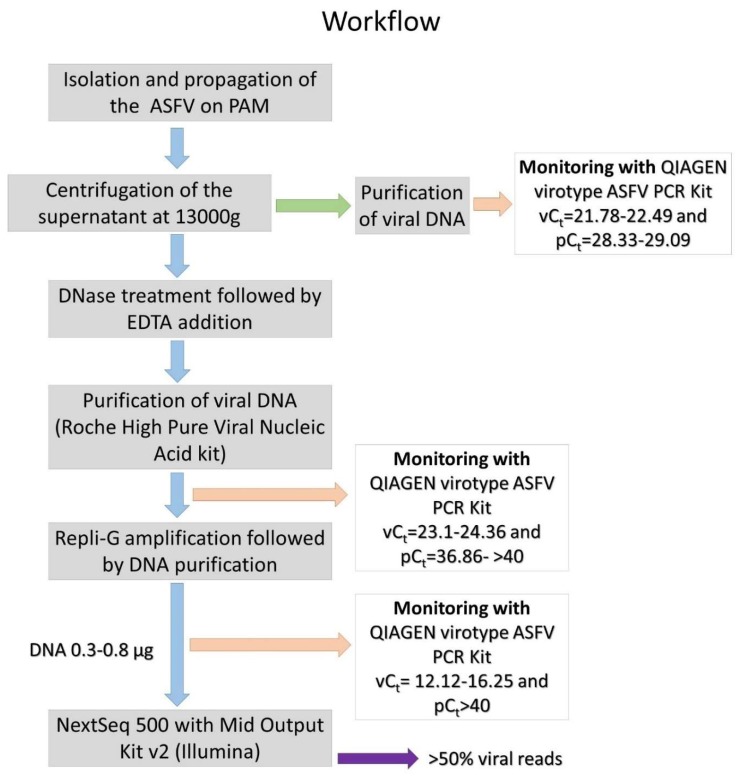
Workflow of the ASFV sequencing protocol. Major steps of the process are depicted together with key values for quality control to ensure successful reproduction. vC_t_, viral genome C_t_ (cycle threshold) value; pC_t_, pig genome C_t_ value.

We developed an ASFV sequencing protocol that gives a simple and effective solution to the common problem of viral DNA shortage and host DNA contamination in ASFV samples. Proper application of the nuclease treatment, whole genome amplification by random PCR, and most importantly, constant monitoring and evaluation of the effectiveness of different phases of sample preparation allowed us to take full advantage of NGS for ASFV genome sequencing. Via sequencing of the first Hungarian ASFV isolate, we highlighted sensitive steps and supplied key reference numbers to assist reproducibility and to facilitate the successful use of this protocol for other ASFV researchers (Figure 5).

## Figures and Tables

**Table 1:** Primers used to amplify three poly C/G regions.

Regions	Primers
14067–14379	>seqASF_14234-F CTGAGATAGCCAAATCAAAATAC
	>seqASF_14234-R CGATTGTAAACTGTATAGTTAATCG
15551–15809	>seqASFV_15670-F CAAAGCAGCCTGTATATGCAATACC
	>seqASFV_15670-R CAATCATTCTATTGTAAACTGTAGAG
19845–20072	>seqASFV_20022-F TAGTACATCAATGTTGTAAGTTTG
	>seqASFV_20022-R CTATCTAAACGTGCTTCTATGAATTC

**Table 2:** Qualifying data for four sequenced samples.

Method	Samples of ASFV_HUN_2018	Viral Reads of the Total (%)	Number of Viral Reads	Mean Coverage	Std. Dev.
Ion PGM System	S43	90.4	179,325	197	129.1
Ion PGM System	S41	87.7	152,865	158	106.5
NextSeq Illumina	S1	50	6,835,057	2557	1624.9
NextSeq Illumina	S20	77	7,115,377	2692	1897.6

**Table 3:** Nucleotide differences between ASFV_HU_2018 and two recent ASFV isolates.

**Differences between ASFV Belgium 2018/1 and ASFV_HUN_2018**				
**Type**	**Mutation**	**Position**	**Localisation**	**Description of Differences**
Indel	deletC	1384.	non-coding region	
Indel	deletT	2956.	non-coding region	
Indel	deletA	12570.	ASFV G ACD 00190 CDS	The ASFV_HUN_2018 contains the “common version” of gene. The adenine insertion is unique in the ASFV Belgium 2018/1.
Indel	delet4C	15670.	MGF 110-13L	The length of this cytosine rich region is variable among isolates.
Indel	delet2G	17845.	non-coding region	
Indel	delet3G	20001.	ASFV G ACD 00350 CDS	The length of this guanine rich region is variable among isolates.
Indel	deletG	21799.	non-coding region	
Point mutation	T->C	26419.	MGF 360-10L	N->S, This nucleotide position is variable among the isolates.
Indel	insT	27422.	non-coding region	
Indel	insT	73257.	non-coding region	
Point mutation	G->A	88348.	C315R	V->I The “common version” of gene contains the codon of valine. The isoleucine is unique in the ASFV_HUN_2018.
Indel	insG	103310.	non-coding region	
Point mutation	T->C	109659.	B263R	This synonym nucleotide change is unique in the ASFV_HUN_2018.
Point mutation	A->G	145065.	D117L	L->P The “common version” of gene contains codon of proline. This amino acid change is unique in the ASFV Belgium 2018/1.
Quasispecies	W (A/T)	190462.	non-coding region	Coverage: 318 Adenine 58%; Thymine 42%
Quasispecies	S (C/G)	190470.	non-coding region	Coverage: 298 Cytosine: 41%, Guanine 59%
**Differences between China/2018/AnhuiXCGQ and ASFV_HUN_2018**				
**Type**	**Mutation**	**Position**	**Localisation**	**Description of Differences**
Indel	insA	1063.	non-coding region	
Indel	insC	1392.	non-coding region	
Indel	ins2C	14235.	MGF 110-14L	The length of this cytosine rich region is variable among isolates.
Indel	ins4G	17631.	non-coding region	
Indel	deletG	17845.	non-coding region	
Indel	delet2G	20001.	ASFV G ACD 00350 CDS	The length of this guanine rich region is variable among isolates.
Point mutation	G->A	88348.	C315R	V->I The “common version” of gene contains the codon of valine. This amino acide is unique in the ASFV_HUN_2018.
Point mutation	T->C	109659.	B263R	This synonym nucleotide change is unique in the ASFV_HUN_2018.
Point mutation	A->G	129413.	O174L	S->P This amino acide change is unique in the China/2018/AnhuiXCGQ
Point mutation	A->G	129517.	O174L	F->S This amino acide change is unique in the China/2018/AnhuiXCGQ
Point mutation	A->G	129542	O174L	S->P This amino acide change is unique in the China/2018/AnhuiXCGQ
Indel	insA	190122.	DP60R	The length of this cytosine rich region is variable among isolates.
Quasispecies	W (A/T)	190462.	non-coding region	Coverage: 318 Adenine 58%; Thymine 42%
Quasispecies	S (C/G)	190470.	non-coding region	Coverage: 298 Cytosine: 41%, Guanine 59%
